# Serum Levels of Proinflammatory Biomarkers in Military Recruits with and without Metabolic Syndrome

**DOI:** 10.1155/2023/4613842

**Published:** 2023-04-30

**Authors:** Abdulrahman K. Al Asmari, Hamoud A. Al Shehri, Haseeb A. Khan, Saud Al Omani, Saeed G. Kadasah, Ghaleb B. Horaib, Ahmed Al Buraidi, Abdullah A. Al Sharif, Fayez S. Mohammed, Rajamohamed Abbasmanthiri, Nasreddien M. Osman

**Affiliations:** ^1^Scientific Research Center, Medical Service Department (MSD), Ministry of Defence, Riyadh, Saudi Arabia; ^2^Adult Cardiology, Prince Sultan Cardiac Center, Medical Service Department (MSD), Ministry of Defence, Riyadh, Saudi Arabia; ^3^Department of Biochemistry, College of Science, King Saud University, Riyadh 11451, Saudi Arabia; ^4^Department of Surgery, Prince Sultan Military Medical City, Medical Service Department (MSD), Ministry of Defence, Riyadh, Saudi Arabia; ^5^Department of Psychiatry, Prince Sultan Military Medical City, Medical Service Department (MSD), Ministry of Defence, Riyadh, Saudi Arabia; ^6^Dermatology Department, Medical Service Department (MSD), Ministry of Defence, Riyadh, Saudi Arabia; ^7^Department of ENT, Prince Sultan Military Medical City, Medical Service Department (MSD), Ministry of Defence, Riyadh, Saudi Arabia; ^8^Department of Dentistry, Prince Sultan Military Medical City, Medical Service Department (MSD), Ministry of Defence, Riyadh, Saudi Arabia; ^9^Prince Sultan Military College of Health Science, Dhahran, Saudi Arabia

## Abstract

**Objectives:**

Inflammatory mediators are associated with many chronic diseases; however, their role in metabolic syndrome (Met-S) is not well documented. We therefore aimed to compare the serum markers of inflammation including C-reactive protein (CRP), myeloperoxidase (MPO), interleukin-6 (IL-6), tumour necrosis factor alpha (TNF-*α*), and TNF-*β* in young military recruits with and without Met-S. We hypothesized that any significant change in inflammatory markers between the two groups would indicate the role of inflammation in Met-S that would help in future directions for screening and treatment of Met-S. *Design and Methods*. A total of 2010 adult men, aged 18-30 years, were divided into two groups: with Met-S (*N* = 488) and without Met-S (*N* = 1522), according to the International Diabetes Federation definition. We compared the serum levels of inflammatory biomarkers between the two groups. We also studied the correlations between the inflammatory markers and the components of Met-S to explore the biomarker potential of inflammatory markers for screening of Met-S. Logistic regression analysis was performed to test the association between inflammatory markers and Met-S.

**Results:**

A large number of subjects in the Met-S group were suffering from obesity. Out of the 2010 total subjects, only 731 (36.4%) had normal fasting blood sugar (FBS), while the prevalence of prediabetes and diabetes was significantly higher in subjects with Met-S. We observed significant increases in serum levels of CRP, MPO, IL-6, and TNF-*β* but not TNF-*α* in subjects with Met-S as compared to subjects without Met-S. All the markers of inflammation showed significant correlations with Met-S, triglycerides (TG), blood pressure, body mass index (BMI), and age; however, none of these markers were correlated with HDL. Logistic regression analysis showed a significant association between Met-S and inflammatory markers.

**Conclusions:**

Serum levels of CRP, MPO, IL-6, and TNF-*β* are significantly increased in young adults with Met-S. This is probably the first study reporting TNF-*β* levels in Met-S. Since a proinflammatory cascade precedes many years before the onset of cardiovascular disease, these inflammatory biomarkers could help in the monitoring of high-risk individuals with Met-S who will be requiring therapeutic intervention.

## 1. Introduction

Metabolic syndrome (Met-S) and associated diseases are a global health problem. Met-S is a cluster of metabolic abnormalities characterized by hypertension, high fasting blood glucose, increased waist circumference, hypertriglyceridemia, and low high-density lipoprotein (HDL) cholesterol levels [1]. In recent years, Met-S is becoming highly prevalent even in young adults [2]. Many of the risk factors associated with Met-S including hypertension, hyperglycaemia, central obesity, and dyslipidemia are preventable and can be controlled by modifications in dietary habits and physical activity, particularly at their early stage [3]. Moreover, uncontrolled hyperglycaemia has also been directly associated with dyslipidemia [4]. Early detection of metabolic modifications that could lead to Met-S would help in the timely consideration of preventive measures such as dietary changes and lifestyle modifications [5].

Modern lifestyle, including sedentary behaviour and high intake of caloric and sugar-rich foods, contributes to metabolic ailments with preceding pathophysiological changes such as hyperglycaemia, obesity, hypertension, dyslipidaemia, and a proinflammatory state associated with accumulation of adipose tissue [6]. Adipose tissue is not just a passive storage of fat but also has roles in immune modulation and inflammatory responses including the secretion of cytokines [7]. Met-S has been independently linked with an inflammatory burden as well as increased oxidative stress [8]. Previous studies have also shown increased secretion of apolipoprotein B, uric acid, fibrinogen, plasminogen activator inhibitor 1, C-reactive protein (CRP), and proinflammatory cytokines in Met-S [7, 9, 10]. Currently, the inflammatory markers are not included in the National Cholesterol Education Program Adult Treatment Panel III (NCEP ATP III) or World Health Organization (WHO) diagnostic criteria for Met-S, but it is most probable that a proinflammatory state is one of the hallmarks of this syndrome.

In this study, we compared serum markers of inflammation including CRP, myeloperoxidase (MPO), interleukin-6 (IL-6), tumour necrosis factor alpha (TNF-*α*), and TNF-*β* in young military recruits with and without Met-S. We also studied the correlations between the inflammatory markers and the components of Met-S to explore the biomarker potential of inflammatory markers for the screening of Met-S. We hypothesized that a clear understanding of the role of inflammatory mediators in Met-S would not only help in selecting potential biomarkers for screening high-risk individuals but also pave the way for exploring therapeutic modalities such as anti-inflammatory drugs for the treatment of Met-S.

## 2. Materials and Methods

### 2.1. Study Population and Medical Observations

A total of 2010 young Saudi men, aged 18-30 years, who applied for recruitment to the Saudi armed forces were included in this study. The study was carried out at the health facility of the selection centers, and all the selected participants individually completed the consent forms. Standardized medical observations including physical examination as well as measurements related to Met-S including blood pressure, waist circumference, height, body weight, and blood biochemistry were performed by trained researchers. Blood pressure was measured using a standard sphygmomanometer one time on each arm in subjects who had been resting for 10 min, and the mean of the two measurements was taken. The complete information of each participant was filled in a specially designed questionnaire based on the guidelines of WHO [11]. The study protocol was approved by the Institutional Research and Ethics Committee.

### 2.2. Inclusion and Exclusion Criteria of Participants

The inclusion criteria were male, Saudi, and young adults in the age range of 18-30 years. The exclusion criteria were the presence of any apparent illness or disability. According to the International Diabetes Federation (IDF) definition (http://www.idf.org) [12], the subjects were considered to have Met-S if they had central obesity (defined as waist circumference >94 cm), plus two of the following four factors: raised fasting plasma glucose >100 mg/dL (5.6 mmol/L) or previously diagnosed type 2 diabetes; systolic BP >130 or diastolic BP >85 mm Hg or treatment of previously diagnosed hypertension; HDL-C <40 mg/dL (1.0 mmol/L) or specific treatment for this lipid abnormality; TG level >150 mg/dL (1.7 mmol/L) or specific treatment for this lipid abnormality.

### 2.3. Biochemical Analysis

Blood samples from each recruitment center were transported to Prince Sultan Military Medical City for biochemical analysis. Blood samples were centrifuged at 1500 × g for 15 min at 4°C; sera were separated and stored at -20°C until analysed. Fasting blood sugar (FBS), total cholesterol, high-density lipoprotein cholesterol (HDL), and triglycerides (TG) were analysed using a Hitachi 902 autoanalyzer. Serum levels of CRP, MPO, IL-6, TNF-*α*, and TNF-*β* were measured using commercially available Quantikine enzyme-linked immunosorbent assay (ELISA) kits (R&D Systems, Bio-Techne Ltd, Abingdon, UK), according to the manufacturer's instructions.

### 2.4. Statistical Analysis

The data were analysed by using the SPSS statistical package version 14 (SPSS Chicago, IL). The chi-square test and Student's *t*-test were used for comparison between the Met-S and without Met-S groups depending on the data type, categorical or continuous, respectively. Pearson and Spearman's tests were used for correlation analysis between various biomarkers and Met-S. Logistic regression analysis was conducted to test the association between inflammatory biomarkers and Met-S. *P* values < 0.05 were considered statistically significant.

## 3. Results

The age of subjects without Met-S was 19.85 ± 2.70 y while those with Met-S were aged 21.14 ± 3.38 y, and this difference was statistically significant (Table 1). Most of the subjects were single (96.2%); however, among married subjects, the majority belonged to the Met-S group. The body weight and BMI were significantly higher in subjects with Met-S as compared to the normal group (Table 1). A large number of subjects in the Met-S group were affected by obesity. Hypertension was prevalent in both groups; however, the frequency of subjects with hypertension was significantly higher in Met-S as compared to subjects without Met-S (Table 1). Out of the 2010 total subjects, only 731 (36.4%) had normal fasting blood sugar (FBS) levels, while the prevalence of prediabetes and diabetes was significantly higher in subjects with Met-S (Table 1).

**Figure 1:**
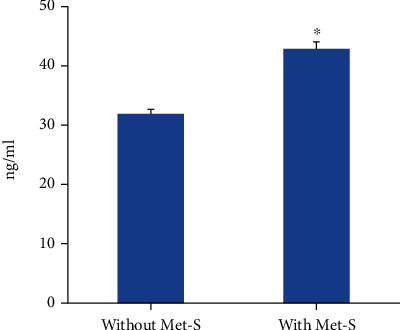
Serum levels of C-reactive protein (CRP) in subjects without Met-S (*N* = 1522) and with Met-S (*N* = 488). ^∗^*P* < 0.001 using *t*-test.

**Figure 2:**
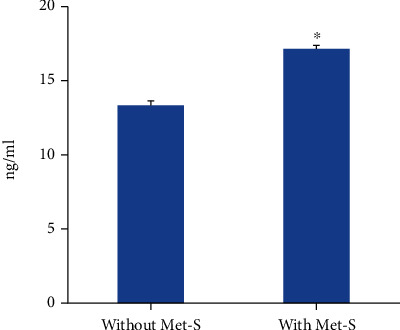
Serum levels of myeloperoxidase (MPO) in subjects without Met-S (*N* = 1522) and with Met-S (*N* = 488). ^∗^*P* < 0.001 using *t*-test.

**Figure 3:**
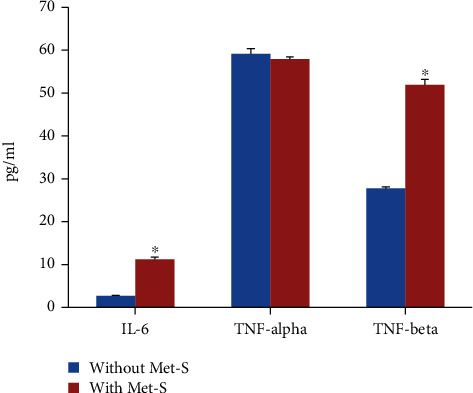
Serum levels of proinflammatory cytokines in subjects without Met-S (*N* = 1522) and with Met-S (*N* = 488). ^∗^*P* < 0.001 using *t*-test.

Serum levels of CRP were significantly higher in subjects with Met-S (42.88 ± 1.13 ng/mL) as compared to subjects without Met-S (31.99 ± 0.69 ng/mL) (Figure 1). The levels of MPO in sera of control subjects were 13.46 ± 0.12 ng/mL, whereas the individuals with Met-S showed significantly higher levels of serum MPO (17.26 ± 0.18 ng/mL) (Figure 2). There was no significant difference in serum levels of TNF-*α* between the two groups. Whereas, serum IL-6 (11.25 ± 0.22 versus 2.73 ± 0.04 pg/mL) and TNF-*β* (52.13 ± 1.21 versus 27.65 ± 0.23 pg/mL) levels were found to be significantly higher in subjects with Met-S as compared to subjects without Met-S (Figure 3). All the markers of inflammation including serum CRP, MPO, IL-6, TNF-*α*, and TNF-*β* showed significant correlations with Met-S, TG, blood pressure, BMI, and age; however, none of these markers were correlated with HDL (Table 2). The results of logistic regression analysis showed a significant association between Met-S and inflammatory markers (Table 3). Compared to other markers of inflammation, IL-6 showed higher odd ratios and therefore more significant association with Met-S as well as other independent variables including BMI, blood pressure, and fasting blood sugar (Table 3).

## 4. Discussion

We observed a high prevalence of obesity and hyperglycemia in subjects with Met-S as compared to subjects without Met-S. Our results also showed a significant increase in inflammatory biomarkers including CRP, MPO, and proinflammatory cytokines in the sera of subjects with Met-S. These markers also showed significant correlations with all the components of Met-S except HDL. A significant association between Met-S and inflammation was also confirmed by logistic regression analysis. The mechanism of a direct relationship between Met-S and inflammation is not fully understood. This can partly be attributed to the stimulation of hepatic CRP production from cytokines, which originate from the adipose tissue; alternatively, insulin resistance can be responsible for the increased production of cytokines [13]. CRP is a sensitive but nonspecific marker of inflammation. High levels of CRP can be predictive markers for Met-S [14, 15] and cardiovascular disease [16, 17]. [18] have suggested that CRP could be used to identify those with risk in developing Met-S.

Myeloperoxidase (MPO) is a marker of neutrophil activation and systemic inflammation. Because MPO can diminish nitric oxide bioavailability that results in endothelial dysfunction [19, 20], it may be an active mediator of atherogenesis [21]. MPO levels have been reported to be higher in patients with coronary artery disease (CAD) and can predict future cardiovascular events, even after correction for traditional risk factors and CRP [22, 23]. Patients with CAD have reflected a strong correlation between increased risk of subsequent cardiovascular events and serum MPO levels [22, 24]. [25] have suggested that inflammatory activation precedes the onset of overt CAD by many years, and elevated MPO levels can predict the future risk of CAD in apparently healthy individuals.

The proinflammatory cytokines, IL-6, TNF-*α*, and TNF-*β*, are secreted during an inflammatory response [7, 26, 27]. The procoagulant cytokine IL-6 is produced by several cell types, and it is a major source of plasma IL-6 [26, 28]. IL-6 plays several roles including acute phase inflammatory reaction, haematopoiesis, immunoglobulins secretion, and T and B cell cooperation function and regulation [26, 29, 30]. Higher levels of IL-6 have been reported in obesity, diabetes, and insulin resistance [26, 31–34]. [18] observed that IL-6 was one of the best markers of CAD risk prediction compared to other inflammatory biomarkers.

In our study, although the serum levels of TNF-*β* were significantly higher in subjects with Met-S compared to subjects without Met-S, there was no difference in TNF-*α* levels between the two groups. The differences in the biological activity between TNF-*α* and TNF-*β* are due to their different abilities for binding to the target cells [35]. The role of TNF-*α* in the development of obesity-related insulin resistance is still controversial. Metabolically triggered inflammation plays an important role in the pathogenesis of obesity-induced insulin resistance and type 2 diabetes mellitus [36, 37]. Previous studies have shown the detrimental effects of TNF-*α* on glucose and lipid metabolism as well as its involvement in *β*-cell apoptosis and endothelial dysfunction in diabetes [38–40]. On contrary, TNF-*α* neutralization in type 2 diabetes patients failed to affect insulin sensitivity [41, 42]. [43] noticed that despite improvements in inflammatory status, chronic TNF-*α* blockage did not improve insulin resistance or endothelial function in volunteers with obesity, insulin resistance, and Met-S.

In conclusion, serum levels of CRP, MPO, IL-6, and TNF-*β* are significantly increased in young adults with Met-S, whereas no significant alteration was observed in TNF-*α*. This is probably the first study reporting TNF-*β* levels in Met-S. We also observed a significantly high prevalence of obesity and hyperglycaemia in subjects with Met-S. An increase in fat tissue in subjects with obesity and/or insulin resistance in subjects with hyperglycaemia would result in low-grade systemic inflammation that could easily be monitored by inflammatory biomarkers well before the onset of cardiovascular disease.

## Figures and Tables

**Table 1:** Characteristics of subjects with and without Met-S.

Parameter	No met-S (*N* = 1522)	Met-S (*N* = 488)	Total (*N* = 2010)	*P* value
Age (years)	19.85 ± 2.70	21.14 ± 3.38	20.16 ± 2.94	<0.001
Age (categorized), *N* (%)				
Age (≤21 y)	1165 (76.5)	283 (58.0)	1448 (72.0)	<0.001
Age (22-26 y)	331 (21.7)	182 (37.3)	513 (25.5)	
Age (>26 y)	26 (1.7)	23 (4.7)	49 (2.4)	
Marital status, *N* (%)				
Single	1476 (97.0)	457 (93.6)	1933 (96.2)	<0.01
Married	46 (3.0)	31 (6.35)	77 (3.8)	
Height (cm)	168.84 ± 6.79	171.04 ± 6.92	169.38 ± 6.89	<0.001
Weight (kg)	69.14 ± 23.07	94.35 ± 14.79	75.26 ± 23.94	<0.001
BMI (kg/m^2^)	24.14 ± 7.36	32.23 ± 3.60	26.10 ± 7.49	<0.001
BMI status (categorized), *N* (%)				
Normal (≤24.9)	855 (56.2)	0 (0.0)	855 (42.5)	<0.001
Overweight (25.0-29.9)	135 (8.9)	91 (18.6)	226 (11.2)	
Obesity (≥30.0)	532 (35.0)	397 (81.4)	929 (46.2)	
Systolic blood pressure, *N* (%)				
≤129.0 (normal)	674 (44.3)	40 (8.2)	714 (35.5)	<0.001
>129.0 (high)	848 (55.7)	448 (91.8)	1296 (64.5)	
Fasting blood sugar (categorized), *N* (%)				
<100.0 (normal)	674 (44.3)	57 (11.7)	731 (36.4)	<0.001
100.0-125.9 (prediabetes)	751 (49.3)	359 (73.6)	1110 (55.2)	
≥126.0 (diabetes)	97 (6.4)	72 (14.8)	169 (8.4)	

Values are presented as number (%) or means ± standard deviation. The chi-square test was used for categorical variables, and an independent *t*-test was used for continuous variables.

**Table 2:** Correlations between serum biomarkers and components of metabolic syndrome.

	CRP	MPO	IL-6	TNF-*α*	TNF-*β*
HDL	*R* = 0.009	*R* = −0.022	*R* = 0.019	*R* = 0.015	*R* = 0.027
	*P* = 0.673	*P* = 0.316	*P* = 0.395	*P* = 0.502	*P* = 0.223
TG	*R* = 0.077	*R* = 0.116	*R* = 0.222	*R* = −0.159	*R* = 0.181
	*P* < 0.01	*P* < 0.001	*P* < 0.001	*P* < 0.001	*P* < 0.001
FBG	*R* = 0.024	*R* = 0.109	*R* = 0.171	*R* = −0.076	*R* = 0.125
	*P* = 0.291	*P* < 0.001	*P* < 0.001	*P* < 0.01	*P* < 0.001
Met-S	*R* = 0.186	*R* = 0.356	*R* = 0.704	*R* = −0.346	*R* = 0.507
	*P* < 0.01	*P* < 0.001	*P* < 0.001	*P* < 0.001	*P* < 0.001
BP	*R* = 0.077	*R* = 0.169	*R* = 0.333	*R* = −0.163	*R* = 0.263
	*P* < 0.01	*P* < 0.001	*P* < 0.001	*P* < 0.001	*P* < 0.001
BMI	*R* = 0.088	*R* = 0.153	*R* = 0.371	*R* = −0.167	*R* = 0.251
	*P* < 0.01	*P* < 0.001	*P* < 0.001	*P* < 0.001	*P* < 0.001
Age	*R* = 0.067	*R* = 0.093	*R* = 0.140	*R* = −0.078	*R* = 0.130
	*P* < 0.01	*P* < 0.01	*P* < 0.001	*P* < 0.01	*P* < 0.001

*R* = correlation coefficient.

**Table 3:** Logistic regression analysis for the association between serum inflammatory markers and metabolic syndrome and other variables.

Biomarkers	Odd ratio (OR)	95% CI	*P* value
Metabolic syndrome			
CRP	1.017	1.013-1.021	<0.01
MPO	1.207	1.176-1.239	<0.001
IL-6	3.967	3.258-4.831	<0.001
TNF-*α*	0.865	0.848-0.882	<0.01
TNF-*β*	1.098	1.087-1.109	<0.01
Body mass index			
CRP	1.006	1.002-1.009	<0.01
MPO	1.064	1.044-1.085	<0.01
IL-6	1.355	1.300-1.412	<0.001
TNF-*α*	0.949	0.935-0.962	<0.01
TNF-*β*	1.044	1.035-1.052	<0.01
Blood pressure			
CRP	1.006	1.002-1.010	<0.01
MPO	1.057	1.036-1.078	<0.01
IL-6	1.174	1.139-1.211	<0.001
TNF-*α*	0.963	0.949-0.977	<0.01
TNF-*β*	1.028	1.020-1.035	<0.01
Fasting blood sugar			
CRP	1.004	1.00-1.008	<0.05
MPO	1.061	1.040-1.082	<0.01
IL-6	1.138	1.108-1.169	<0.001
TNF-*α*	0.956	0.942-0.970	<0.01
TNF-*β*	1.025	1.018-1.032	<0.01

## Data Availability

Data are available from the corresponding author upon reasonable request.

## References

[1] NCEP (2002). Third report of the National Cholesterol Education Program (NCEP) expert panel on detection, evaluation, and treatment of high blood cholesterol in adults (adult treatment panel III). Final report. *Circulation*.

[2] Al-Shehri H. A., Al-Asmari A. K., Khan H. A. (2021). Recent trends of metabolic syndrome and its components in military recruits from Saudi Arabia. *Medicine*.

[3] Al-Shehri H. A., Al-Asmari A. K., Khan H. A. (2022). Association between preventable risk factors and metabolic syndrome. *Open Medicine*.

[4] Khan H. A. (2007). Clinical significance of HbA1c as a marker of circulating lipids in male and female type 2 diabetic patients. *Acta Diabetologica*.

[5] Sostaric A., Jenko B., Kozjek N. R. (2019). Detection of metabolic syndrome burden in healthy young adults may enable timely introduction of disease prevention. *Archives of Medical Science*.

[6] James A. M., Collins Y., Logan A., Murphy M. P. (2012). Mitochondrial oxidative stress and the metabolic syndrome. *Trends in Endocrinology & Metabolism*.

[7] Lee Y. H., Pratley R. E. (2005). The evolving role of inflammation in obesity and the metabolic syndrome. *Current Diabetes Reports*.

[8] Van Guilder G. P., Hoetzer G. L., Greiner J. J., Stauffer B. L., DeSouza C. A. (2006). Influence of metabolic syndrome on biomarkers of oxidative stress and inflammation in obese adults. *Obesity*.

[9] Eckel R. H., Grundy S. M., Zimmet P. Z. (2005). The metabolic syndrome. *The Lancet*.

[10] Gustafson B., Hammarstedt A., Anderson C. X., Smith U. (2007). Inflamed adipose tissue. *Arteriosclerosis, Thrombosis, and Vascular Biology*.

[11] WHO (2013). *A Global Brief on Hypertension Silent Killer: Global Public Health Crisis*.

[12] International Diabetes Federation The IDF consensus worldwide definition of the metabolic syndrome. http://www.idf.org/metabolic_syndrome.

[13] Sutherland J., McKinnley B., Eckel R. H. (2004). The metabolic syndrome and inflammation. *Metabolic Syndrome and Related Disorders*.

[14] Hassinen M., Lakka T., Komulainen P., Gylling H., Nissinen A., Rauramaa R. (2006). C-reactive protein and metabolic syndrome in elderly women. *Diabetes Care*.

[15] Sur G., Floca E., Kudor-Szabadi L., Sur M. L., Sur D., Samasca G. (2014). The relevance of inflammatory markers in metabolic syndrome. *Maedica*.

[16] Khan H. A., Alhomida A. S., Sobki S. H., Al M. A. (2012). Significant increases in monocyte counts and serum creatine kinase in acute myocardial infarction versus general infections. *Indian Journal of Pathology and Microbiology*.

[17] Khan H. A., Alhomida A. S., Sobki S. H. (2013). Lipid profile of patients with acute myocardial infarction and its correlation with systemic inflammation. *Biomarker Insight*.

[18] Rao V. S., Nagaraj R. K., Hebbagodi S., Kadarinarasimhiah N. B., Kakkar V. V. (2010). Association of inflammatory and oxidative stress markers with metabolic syndrome in Asian Indians in India. *Cardiology Research and Practice*.

[19] Abu-Soud H. M., Hazen S. L. (2000). Nitric oxide is a physiological substrate for mammalian peroxidases. *The Journal of Biological Chemistry*.

[20] Vita J. A., Brennan M. L., Gokce N. (2004). Serum myeloperoxidase levels independently predict endothelial dysfunction in humans. *Circulation*.

[21] Nicholls S. J., Hazen S. L. (2005). Myeloperoxidase and cardiovascular disease. *Arteriosclerosis, Thrombosis, and Vascular Biology*.

[22] Baldus S., Heeschen C., Meinertz T. (2003). Myeloperoxidase serum levels predict risk in patients with acute coronary syndromes. *Circulation*.

[23] Zhang R., Brennan M. L., Fu X. (2001). Association between myeloperoxidase levels and risk of coronary artery disease. *JAMA*.

[24] Gómez García A., Rivera Rodríguez M., Gómez Alonso C., Rodríguez Ochoa D. Y., Alvarez A. C. (2015). Myeloperoxidase is associated with insulin resistance and inflammation in overweight subjects with first-degree relatives with type 2 diabetes mellitus. *Diabetes and Metabolism Journal*.

[25] Meuwese M. C., Stroes E. S. G., Hazen S. L. (2007). Serum myeloperoxidase levels are associated with the future risk of coronary artery disease in apparently healthy individuals: the EPIC-Norfolk prospective population study. *Journal of the American College of Cardiology*.

[26] Kir S., Ekiz K., Alacam H., Turkel R., Koroglu E., Altintop B. L. (2019). The association between pro and anti-inflammatory markers with the components of metabolic syndrome. *Acta Endocrinologica (Bucharest)*.

[27] Trayhurn P. (2005). Endocrine and signalling role of adipose tissue: new perspectives on fat. *Acta Physiologica Scandinavica*.

[28] Fried S. K., Bunkin D. A., Greenberg A. S. (1998). Omental and subcutaneous adipose tissues of obese subjects release interleukin-6: depot difference and regulation by glucocorticoid. *The Journal of Clinical Endocrinology and Metabolism*.

[29] Ridker P. M., Willerson J. T. (2004). Inflammation as a cardiovascular risk factor. *Circulation*.

[30] Santos-Rosa M., Bienvenu J., Whicher J., Burtis C. A., Ashwood E. R. (1999). Cytokines. *Tietz Textbook of Clinical Chemistry*.

[31] Kershaw E. E., Flier J. S. (2004). Adipose tissue as an endocrine organ. *The Journal of Clinical Endocrinology and Metabolism*.

[32] Kristiansen O. P., Mandrup-Poulsen T. (2005). Interleukin-6 and diabetes. *Diabetes*.

[33] Rajala M. W., Scherer P. E. (2003). Minireview: the adipocyte- at the crossroads of energy homeostasis, inflammation and atherosclerosis. *Endocrinology*.

[34] Vozarova B., Weyer C., Hanson K., Tataranni P. A., Bogardus C., Pratley R. E. (2001). Circulating interleukin-6 in relation to adiposity, insulin action, and insulin secretion. *Obesity Research*.

[35] Kircheis R., Milleck J., Korobko V. G., Shingarova L. N., Schmidt H. E. (1992). Differences in the biological activity of TNF alpha and TNF beta correlate with their different abilities for binding to the target cells. *European Cytokine Network*.

[36] Bays H. E., González-Campoy J. M., Bray G. A. (2008). Pathogenic potential of adipose tissue and metabolic consequences of adipocyte hypertrophy and increased visceral adiposity. *Expert Review of Cardiovascular Therapy*.

[37] Pradhan A. (2007). Obesity, metabolic syndrome, and type 2 diabetes: inflammatory basis of glucose metabolic disorders. *Nutrition Reviews*.

[38] Dandona P., Weinstock R., Thusu K., Abdel-Rahman E., Aljada A., Wadden T. (1998). Tumor necrosis factor alpha in sera of obese patients: fall with weight loss. *The Journal of Clinical Endocrinology and Metabolism*.

[39] Hotamisligil G. S. (1999). The role of TNF*α* and TNF receptors in obesity and insulin resistance. *Journal of Internal Medicine*.

[40] Zhang S., Kim K. H. (1995). TNF-alpha inhibits glucose-induced insulin secretion in a pancreatic beta-cell line (INS-1). *FEBS Letters*.

[41] Dominguez H., Storgaard H., Rask-Madsen C. (2005). Metabolic and vascular effects of tumor necrosis factor-alpha blockade with etanercept in obese patients with type 2 diabetes. *Journal of Vascular Research*.

[42] Paquot N., Castillo M. J., Lefèbvre P. J., Scheen A. J. (2000). No increased insulin sensitivity after a single intravenous administration of a recombinant human tumor necrosis factor receptor: Fc fusion protein in obese insulin-resistant patients. *The Journal of Clinical Endocrinology and Metabolism*.

[43] Wascher T. C., Lindeman J. H. N., Sourij H., Kooistra T., Pacini G., Roden M. (2011). Chronic TNF-*α* neutralization does not improve insulin resistance or endothelial function in “healthy” men with metabolic syndrome. *Molecular Medicine*.

